# Circular RNA circ-MAT2B facilitates glycolysis and growth of gastric cancer through regulating the miR-515-5p/HIF-1α axis

**DOI:** 10.1186/s12935-020-01256-1

**Published:** 2020-05-16

**Authors:** Jia Liu, Haiying Liu, Qingshan Zeng, Pei Xu, Mingxing Liu, Ning Yang

**Affiliations:** Department of Nutriology, The Third Affiliated Hospital of Guangzhou Medical University, 63 Duobao Road, Liwan District, Guangzhou, 510000 China

**Keywords:** circ-MAT2B, miR-515-5p, HIF-1α, Glycolysis

## Abstract

**Background:**

Circular RNAs (circRNAs) are a special kind of non-coding RNAs that are implicated in cancer malignant behavior, including glycolysis. However, their contributions to gastric cancer (GC) cell glycolysis are still poorly understood. In the present study, we aimed to investigate the glycolysis-related role of circ-MAT2B in GC.

**Methods:**

Gene expression was determined by qRT-PCR analysis. Protein level was detected by Western blot. The CCK-8, colony and EdU assays were carried out to assess GC cell viability, colony formation and DNA synthesis rate. Glycolysis was determined by glucose uptake and lactate production. The positive regulatory network of circ-MAT2B/miR-515-5p/HIF-1α was identified by RNA pull-down, RIP, ChIP and luciferase reporter assays. The in vivo role of circ-MAT2B was evaluated by using xenograft tumor model.

**Results:**

Circ-MAT2B was notably increased in GC and could be used as a sensitive and specific indicator of GC diagnosis and prognosis. Stable knockdown of circ-MAT2B dramatically inhibited GC cell viability, colony formation, DNA synthesis, glucose uptake and lactate production in vitro, and retarded tumor growth in vivo. Mechanistically, circ-MAT2B was dominantly located in the cytoplasm and acted as a ceRNA to sponge miR-515-5p and increase HIF-1α expression. Silencing of miR-515-5p or overexpression of HIF-1α could evidently rescue the attenuated aggressive phenotype of GC cells caused by circ-MAT2B knockdown. Importantly, HIF-1α was able to directly bind to circ-MAT2B promoter and transcriptionally activate circ-MAT2B, thus forming a positive feedback loop.

**Conclusion:**

Our data suggest that circ-MAT2B is a oncogenic circRNA in GC and provide a promising therapeutic target for GC patients.

## Background

Gastric cancer (GC) is a major public health problem worldwide and is responsible for more than 1 million new cases and an estimated 783,000 deaths in 2018, enabling it the fifth most commonly diagnosed cancer and the third leading cause of cancer-related death [1, 2]. The etiology and pathogenesis of GC have not been fully elucidated, involving many risk factors, such as helicobacter pylori infection, genetic factors, history of gastric disease, living habits and diet, psychological factor and so on [3]. Therefore, it is urgent to identify the key regulatory molecules that affect the evolution and progression of GC, so that we can better understand and control it.

Recently, the field of non-coding RNA has attracted a lot of attention, especially circular RNA (circRNA). CircRNA is derived from an intriguing and specialized form of alternative splicing in which the 3′-tail of an exon backsplices and joins the 5′-head of the exon localized up-stream, thus forming a covalent closed loop structure [4, 5]. It is highly resistant to exonuclease, which makes it very stable and allows it to play a longer lasting role in the body than other conventional linear RNAs [6]. Emerging evidence shows that circRNA is closely implicated in human development and diseases [7].

microRNAs (miRNAs) are a class of endogenous non-coding single stranded RNA molecules with a length of about 22 nucleotides that participate in the phenotype of cancer cells [8, 9]. Up to now, extensive deregulated circRNAs have been identified in various human cancers [10], and they functioned as oncogenes or tumor suppressors mainly through acting as a competing endogenous RNA (ceRNA) to abundantly sponge miRNAs and reduce the inhibitory effects of miRNAs on their target genes [11, 12]. Fox instance, circ-HIPK3 was shown to be upregulated in human colorectal cancer and promoted tumorigenesis and progression via sponging miR-7 and increasing the levels a series of protooncogenes [13]. And circ-ATXN7 was significantly increased in gastric cancer and expedited tumor growth via absorbing miR-4319 and elevating ENTPD4 [14].

Of note, a novel circRNA, circ-MAT2B, has recently been reported as a key promoter of hepatocellular carcinoma malignancy [15]. Nevertheless, its role in GC is still unclear. Here, we characterized the expression level, biological function as well as clinical implication of circ-MAT2B in GC, and also further deciphered the underlying the mechanism of its action.

## Materials and methods

### Tissues, cell lines and mice

A total of 120 pairs of fresh-frozen GC and paracancerous normal tissues were obtained at the time of surgery at The Third Affiliated Hospital of Guangzhou Medical University. And the tissues were immediately frozen in liquid nitrogen and stored at − 80 °C. All patients did not receive preoperative radiotherapy or chemotherapy and provided written informed consent prior to tissue collection. Besides, plasma samples from 36 healthy controls and 40 GC patients were also collected for testing the diagnostic value of circ-MAT2B in GC. Two GC cell lines (AGS and MKN45) were purchased from ATCC and cultured in DMEM medium supplemented with 10% fetal bovine serum. For animal experiment, 4–6 weeks old male Balb/c nude mice were used and they were divided into two groups, three in each group. And 1 × 10^6^ AGS cells were subcutaneously injected into nude mice to establish the xenograft model. After 5 weeks, mice were sacrificed and tumor tissues were collected for subsequent assays. This study was conducted in accordance with the guideline of the Ethical and Scientific Committees of The Third Affiliated Hospital of Guangzhou Medical University.

### RNA extraction and qRT-PCR

The total RNA was isolated from GC tissues and cells by using TRIzol reagent (Invitrogen, CA, USA) following manufacturer’s recommendations. And plasma RNA was extracted by BIOG cfRNA Easy Kit (BIODAI, Changzhou, China) as per standard protocol. Then, 1 μg RNA was reverse transcribed into cDNA by using Taqman RT kit (Applied Biosciences, CA, USA), followed by amplification and quantification using Power SYBR Green qPCR Master Mix (Applied Biosciences). For detecting miRNA, the TaqMan MicroRNA Reverse Transcription kit (Applied Biosystems) was used to reverse transcribe, and TaqMan 2 × Universal PCR Master Mix No AmpErase UNG (Applied Biosystems) was used to quantify. The 2^−ΔΔCt^ formula was applied to determine RNA relative expression level. GAPGH and U6 were respectively used as control references for circRNA/mRNA and miRNA. The primer sequences are as follows:

Circ-MAT2B: Forward: 5′-GATCACTGGCAGCAGAGGTT-3′, Reverse: 5′-CAGTGGCACCAGTAACCAGA-3′; HIF-1α: Forward: 5′-GAAAGCGCAAGTCCTCAAAG-3′, Reverse: 5′-TGGGTAGGAGATGGAGATGC-3′; GAPDH: Forward: 5′-AGCCACATCGCTCAGACAC-3′, Reverse: 5′-GCCCAATACGACCAAATCC-3′.

### Subcellular fractionation and fluorescence in situ hybridization (FISH)

The RNA in the cytoplasm and nucleus was extracted by using Nuclear/cytoplasmic fractionation PARIS Kit (Life Technologies, CA, USA) following manufacturer’s recommendations. GAPDH and U6 were used as cytoplasmic and nucleus reference fragments, respectively. FISH assay was carried out by using RNAscope Multiplex Assay Kit (Thermo Fisher Scientific, MA, USA) according to manufacturer’s instructions, the fluorescence signal was then observed with microscopy.

### Construction of stable circ-MAT2B knockdown cell lines

Two shRNAs targeting the junction site of circ-MAT2B were designed (sh-circ-MAT2B#1 and sh-circ-MAT2B#2) and inserted into pLV-EF1a-EGFP(2A)-Puro vector, followed by lentivirus package and infection into AGS and MKN45 cell lines with 6 μg/ml polybrene (Sigma-Aldrich, MO, USA). Then, the stable circ-MAT2B knockdown cells were selected in the presence of 1.5 μg/ml puromycin (Sigma-Aldrich).

### Cell transfection

HIF-1α-overexpressing pcDNA 3.0 plasmid (Invitrogen) and HIF-1α siRNA (RiboBio Guangzhou, China) were generated and transfected into AGS and MKN45 cells at a final concentration of 2 ng and 10 nM, respectively. The process of transfection was conducted by using Lipofectamine 3000 reagent (Invitrogen) as per the standard protocols.

### CCK-8, colony formation and EdU assays

For CCK-8 assay, 2 × 10^3^ AGS and MKN45 cells plated onto 96-wells plates and cultured for the indicated time, followed by addition into 10 μl CCK-8 solution (Dojindo, Kumamoto, Japan) and incubation for 2.5 h at 37 °C, then the absorbance at 450 nm was measured. For colony formation, cells were cultured in 6-well plates for 14 days and stained with crystal violet, followed by calculation of the number of clones. DNA synthesis rate was tested by Click-iT EdU Imaging Kits (Invitrogen) according to manufacturer’s instructions.

### Analysis of glycolysis

2 × 10^3^ AGS and MKN45 cells plated onto 96-wells plates, and glycolysis was determined by glucose uptake and lactate production with Glucose Assay Kit (#ab65333, Abcam) and Lactate Assay Kit (#ab65330, Abcam) following manufacturer’s recommendations.

### RNA immunoprecipitation (RIP)

RIP assay was carried out by using the Magna RIP RNA-Binding Protein Immunoprecipitation Kit (Millipore, MA, USA). In brief, AGS and MKN45 cells were UV-crosslinked with 100,000 μJ/cm^2^ twice and lysed in RIP lysis buffer, followed by incubation with anti-Ago2 antibody (#ab57113, Abcam) and protein G magnetic beads. After six washes, the immunocomplexes bound by Ago2 were eluted and incubated with proteinase K at 55 °C for 30 min to digest the proteins, followed by RNA extraction and PCR analysis.

### Biotin-coupled probe pull-down assay

AGS and MKN45 cell lysates were collected using RIPA buffer plus RNase inhibitor (Promega), followed by incubation with the biotin-labeled RNA probe targeting the junction site of circ-MAT2B designed and synthesized by RiboBio at 37 °C for 2 h. Then, the washed streptavidin magnetic beads (Invitrogen) were added into above lysates and incubated for 30 min at 37 °C. After six washes, the immunocomplexes bound by circ-MAT2B probe were eluted for RNA extraction and qRT-PCR analysis.

### Luciferase reporter assay

2 × 10^3^ AGS and MKN45 cells plated onto 96-wells plates and allowed to settle overnight. The constructed luciferase plasmids (Luc-circ-MAT2B-WT, Luc-circ-MAT2B-Mut#1, Luc-circ-MAT2B-Mut#2, WT-Luc, S1-Mut-Luc and S2-Mut-Luc) plus 5 ng pRL-TK-Renilla were respectively transfected into AGS and MKN45 cells by using Lipofectamine 3000 reagent (Invitrogen). 48 h later, the luciferase activity was detected by using a commercial kit (Promega, WI, USA).

### Western blot

The total protein from GC cells and transplanted tumors was isolated by using RIPA buffer supplemented with protease inhibitor cocktail, followed by addition into loading dye and boil for 5 min. 10 μg protein was separated on the 10% SDS-PAGE gel, transferred onto PVDF membrane and blocked with 5% defatted milk powder. Then, the blot was incubated with anti-HIF-1α (#ab2185, Abcam) and anti-GAPDH (#sc-47,724, Santa Cruz Biotechnology) primary antibodies and goat derived IgG H&L secondary antibodies, respectively. The immunoreactive bands were visualized by enhanced chemiluminescence ECL kit and analyzed by Quantity One system (Bio-Rad, CA, USA).

### Chromatin immunoprecipitation (ChIP)

ChIP assay was conducted by using SimpleChIP^®^ Plus Enzymatic Chromatin IP Kit (Cell Signaling Technology, MA, USA) following manufacturer’s recommendations. In short, AGS and MKN45 cells were crosslinked with 1% formaldehyde and terminated with 0.125 M glycine. Then, cells were collected for sonication to yield chromatin fragments with an average size of 200 ~ 1000 bp, followed by incubation with anti-HIF-1α (#ab2185, Abcam) antibody at 4 °C overnight and agarose beads at 37 °C for 1 h with rotation. After four washes, the immunocomplexes were elute for DNA extraction and qPCR analysis. The primer sequences are as follows:

Site 1: Forward: 5`-AGTGAGCCGAGATCATGTCA-3`, Reverse: 5`-TTAGCCAGGATGGTCTCGAT-3`. Site 2: Forward: 5`-ATAGCGGTGCAAAGCTCACT-3`, Reverse: 5`-AGCGGCCTTAAGTTTCACAA-3`.

### Statistic analysis

The SPSS v23 software was used to perform all statistical analysis. The comparison between the two groups was performed by Student’s *t* test, and the correlations between circ-MAT2B and GC patient clinical features were determined by Chi square test. Receiver Operating Characteristic (ROC) curve and Kaplan–Meier plotter were used to measure the diagnostic and prognostic utility of circ-MAT2B in GC. *P* value ≤ 0.05 was considered significant and two-sided unless otherwise specified.

## Results

### Circ-MAT2B is significantly overexpressed in GC

**Fig. 1:**
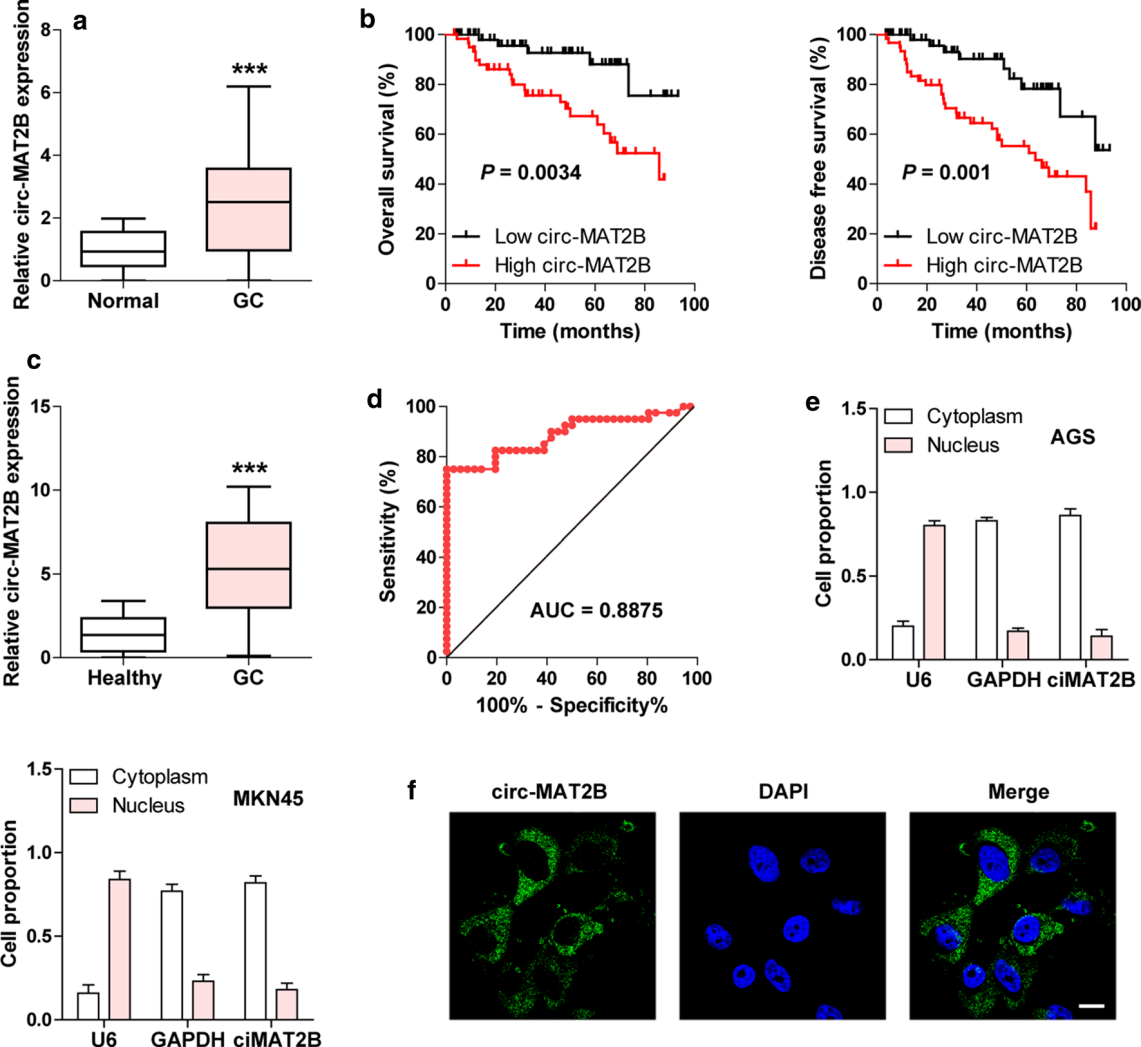
Circ-MAT2B is significantly increased in GC. **a** qRT-PCR analysis of circ-MAT2B in GC and adjacent normal tissues. **b** The survival curves of GC patients with low or high circ-MAT2B expression. **c** qRT-PCR analysis of circ-MAT2B in plasma samples from GC patients and healthy controls. **d** ROC curve detecting the diagnostic utility of plasma circ-MAT2B for GC patients. (**e, f**) qRT-PCR analysis of the subcellular localization of circ-MAT2B in GC cells. DAPI was used to stain nucleus. Scale bar = 20 μm, ***p < 0.001

We first collected 120 paired GC and adjacent normal tissues to detect circ-MAT2B expression. The qRT-PCR results showed that circ-MAT2B was notably upregulated in GC tissues (Fig. 1a). Then, we evaluated the correlations between circ-MAT2B expression and clinicopathological features of GC patients, as shown in Table 1, high circ-MAT2B expression was positively correlated with larger tumor size, lymph node metastasis as well as advanced TNM stage, and patients with high circ-MAT2B had shorter overall and disease-free survival time than those with low circ-MAT2B (Fig. 1b). Furthermore, high circ-MAT2B expression was also observed in GC plasma samples (Fig. 1c), and the area under ROC curve (AUC) was 0.8875 (95% confidence interval: 0.8106 to 0.9644) (Fig. 1d), hinting its good diagnostic performance. In addition, qRT-PCR and FISH results showed that circ-MAT2B was preferentially localized in the cytoplasm (Fig. 1e, f). These data suggest that circ-MAT2B is a dysregulated circRNA in GC and may play important functional roles.

**Table 1:** The correlations between circ-MAT2B expression and clinicopathological features in GC patients (n = 120)

Parameters	All cases	CIRC-MAT2B expression		*P* value
		Low	High	
Gender				
Male	98	48	50	0.637
Female	22	12	10	
Age (years)				
≤ 60	48	23	25	0.709
> 60	72	37	35	
Tumor size				
≤ 5	69	44	25	0.000
> 5	51	16	35	
Lymph node metastasis				
No	54	34	20	0.01
Yes	66	26	40	
TNM stage				
I–II	50	32	18	0.016
III–IV	70	28	42	
Differentiation grade				
Well-moderate	68	37	31	0.269
Poor-undifferentiation	52	23	29	

### Knockdown of circ-MAT2B inhibits GC cell proliferation and glycolysis

**Fig. 2:**
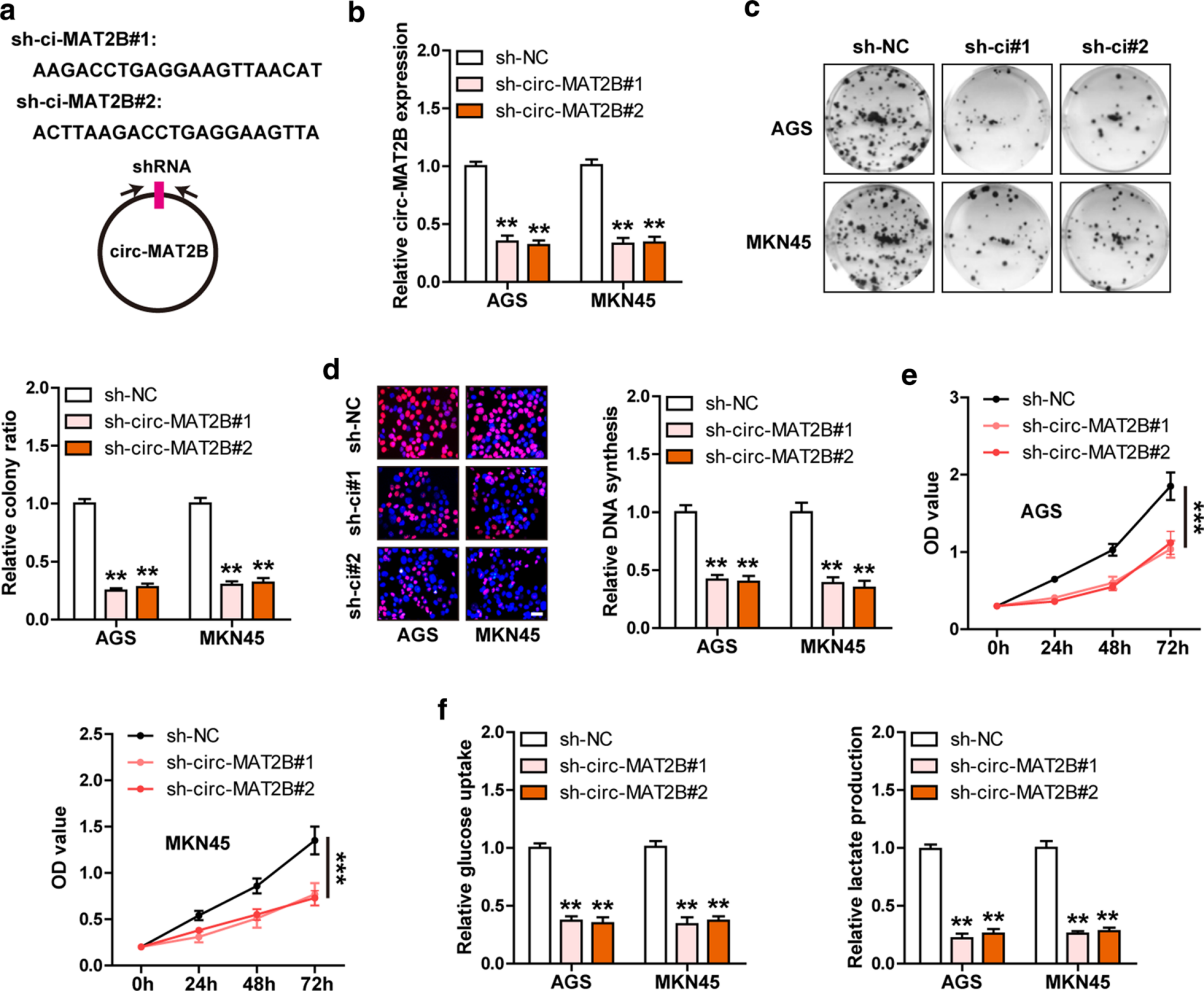
Knockdown of circ-MAT2B weakens GC cell proliferation and glycolysis. **a** The sketch showing two shRNAs targeting the junction sequence of circ-MAT2B. **b** qRT-PCR analysis verifying the silencing effect of above two shRNAs. (**c**–**e**) Colony formation, EdU and CCK-8 assays detecting the proliferation of AGS and MKN45 cells after circ-MAT2B depletion. **f** The level of glycolysis determined by glucose uptake and lactate production in AGS and MKN45 cells after circ-MAT2B depletion. Scale bar = 20 μm, **p < 0.01, ***p < 0.001

Subsequently, we designed two shRNAs targeting the junction site of circ-MAT2B (Fig. 2a) and generated stable circ-MAT2B knockdown AGS and MKN45 cell lines (Fig. 2b). The colony formation assays showed that depletion of circ-MAT2B resulted in a significant decrease in the number of clones (Fig. 2c). And the DNA synthesis rate was evidently slowed in circ-MAT2B-silenced GC cells as compared to control cells (Fig. 2d). Similarly, cell viability was significantly weakened after knockdown of circ-MAT2B (Fig. 2e). Besides, we observed that circ-MAT2B also affected GC cell glycolysis, in which circ-MAT2B knockdown led to a sharp decrease in glucose uptake and lactate production (Fig. 2f). These functional data indicate that circ-MAT2B is a promoter of GC cell malignant phenotype.

### Circ-MAT2B acts as a ceRNA to sponge miR-515-5p

**Fig. 3:**
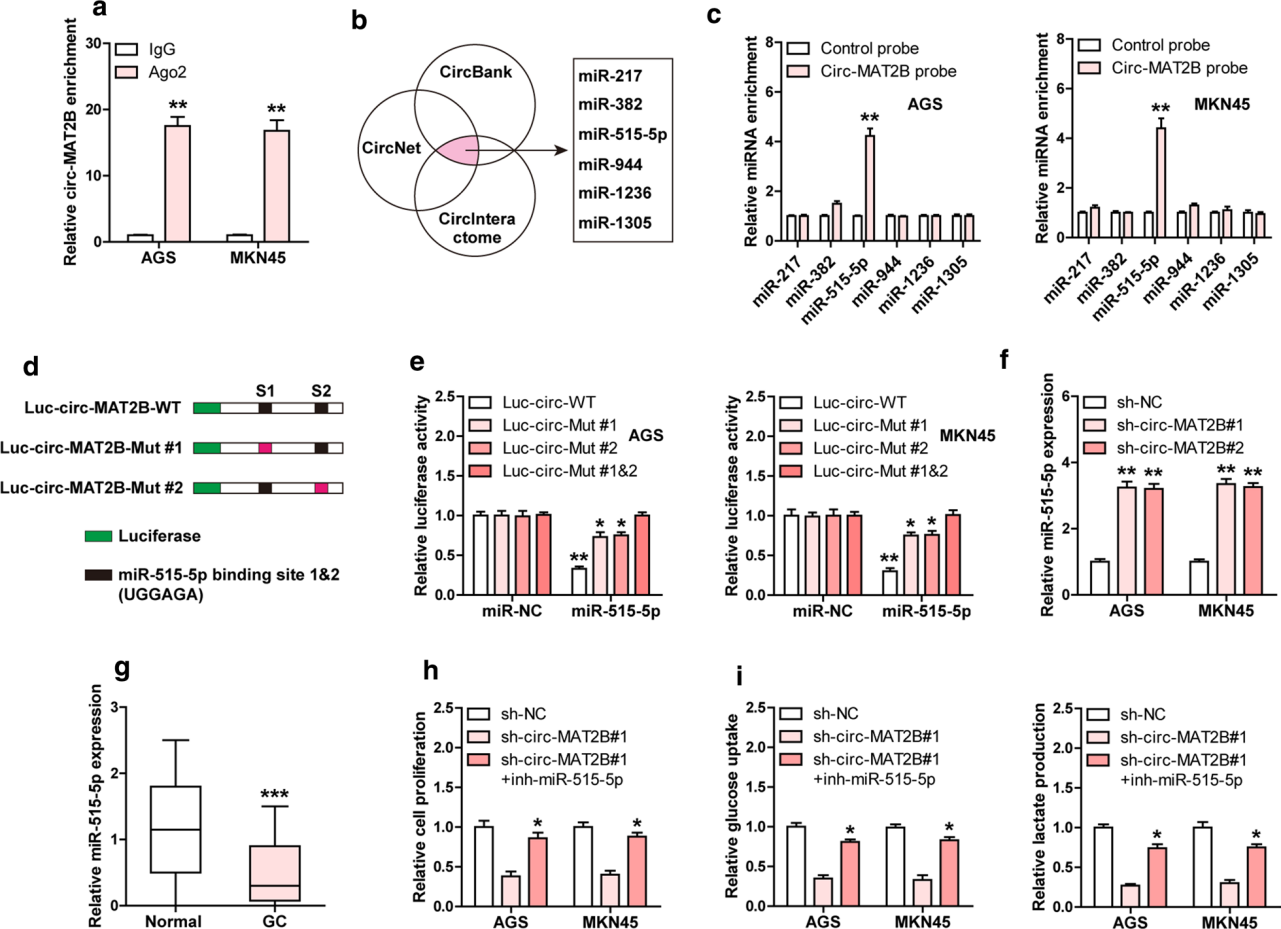
Circ-MAT2B sponges miR-515-5p in GC cells. **a** RIP assay in AGS and MKN45 cells using anti-Ago2 antibody, followed by qRT-PCR analysis of circ-MAT2B expression. **b** The indicated three online tools predicting six miRNAs bound by circ-MAT2B. **c** RNA pull-down in AGS and MKN45 cells using biotin-labeled circ-MAT2B probe, followed by qRT-PCR analysis. **d** The sketch showing the luciferase reporter assay using wild-type or mutant circ-MAT2B vector. **e** The luciferase reporter assay in AGS and MKN45 cells co-transfected with miR-515-5p mimics and the indicated luciferase circ-MAT2B vector. **f**, **g** qRT-PCR analysis of miR-515-5p expression in circ-MAT2B knockdown GC cells or in GC tissues. (**h**, **i**) The indicated functional assays in circ-MAT2B knockdown GC cells transfected with miR-515-5p inhibitors. *p < 0.05, **p < 0.01, ***p < 0.001

In light of the cytoplasmic localization of circ-MAT2B in GC cells, we speculated that it may function as a ceRNA to sponge miRNAs. As expected, the RIP results showed that circ-MAT2B was abundantly enriched by Ago2 (Fig. 3a), a member of RNA-induced silencing complex (RISC) required for miRNA-mediated gene silencing [16], implying that circ-MAT2B may function by miRNA. Then, we analyzed three online tools (CircBank: http://www.circbank.cn/ [17], CircNet: http://circnet.mbc.nctu.edu.tw/ [18], CircInteractome: https://circinteractome.nia.nih.gov/ [19])and found that six miRNAs including miR-217, miR-382, miR-515-5p, miR-944, miR-1236 and miR-1305 may bind to circ-MAT2B (Fig. 3b). To verify this prediction, we performed biotin-coupled RNA pull-down assay and the results showed that only miR-515-5p was significantly enriched by circ-MAT2B in both AGS and MKN45 cells (Fig. 3c). There are two predicted miR-515-5p binding site on circ-MAT2B (Additional file 1: Figure S1), and we mutated them to conduct luciferase reporter assay (Fig. 3d), the results showed that miR-515-5p overexpression evidently decreased the luciferase activity of wild-type vector, while this effect was partly blocked after mutation of miR-515-5p binding site 1 or 2, and was wholly abolished after mutation of both (Fig. 3e). Besides, knockdown of circ-MAT2B resulted in a substantial increase of miR-515-5p expression level (Fig. 3f), and miR-515-5p was significantly downregulated in GC tissues in comparison to normal tissues (Fig. 3g). Moreover, the attenuated GC cell proliferation and glycolysis caused by circ-MAT2B were effectively rescued after silencing of miR-515-5p in both AGS and MKN45 cells (Fig. 3h, i). These results demonstrate that circ-MAT2B is able to sponge and inhibit miR-515 in GC.

### HIF-1α is the downstream target of the circ-MAT2B/miR-515-5p regulatory axis

**Fig. 4:**
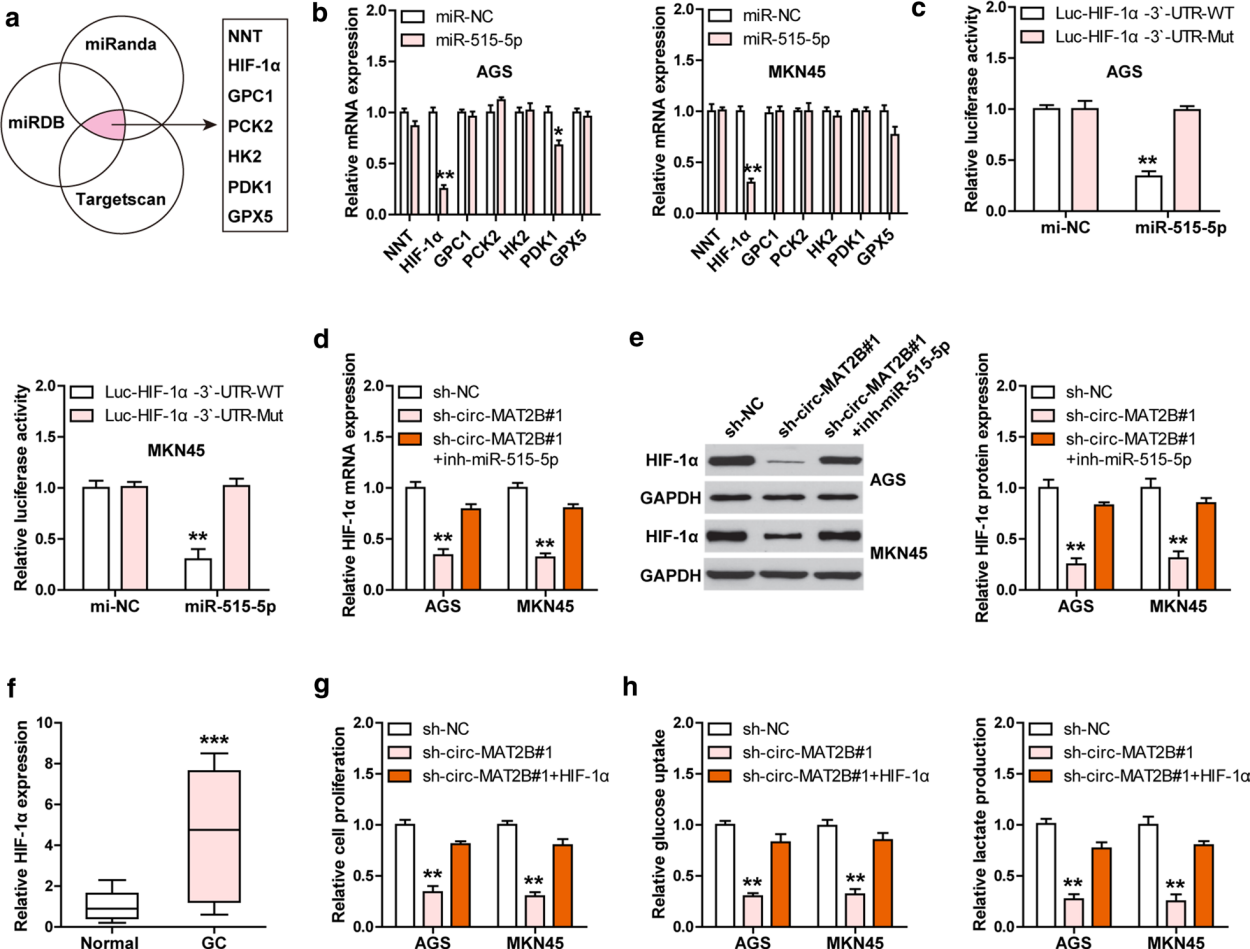
Circ-MAT2B sponges miR-515-5p and upregulates HIF-1α. **a** The indicated three online tools predicting seven genes bound by miR-515-5p. **b** qRT-PCR analysis of seven gene levels in miR-515-5p-overexpressing AGS and MKN45 cells. **c** The luciferase reporter assay in AGS and MKN45 cells co-transfected with miR-515-5p mimics and the indicated luciferase HIF-1α-3`-UTR vector. **d**, **e** The expression level of HIF-1α in circ-MAT2B-depleted GC cells transfected with miR-515-5p inhibitors. **f** qRT-PCR analysis of HIF-1α in GC and normal tissues. **g**, **h** The indicated functional assays in circ-MAT2B knockdown GC cells transfected with HIF-1α-overexpreesing plasmid. **p < 0.01, ***p < 0.001

To find the downstream genes of miR-515-5p, we analyzed three online tools (miRanda: http://www.microrna.org/microrna/home.do [20], miRDB: http://www.mirdb.org/ [21], Targetscan: http://www.targetscan.org/vert_72/ [22]) and focused on the glycolysis-related genes. A total of seven genes were predicted to be bound by miR-515-5p, including NNT, HIF-1α, GPC1, PCK2, HK2, PDK1 and GPX5 (Fig. 4a). The qRT-PCR results showed that only HIF-1α level was affected after miR-515-5p overexpression in both AGS and MKN45 cells (Fig. 4b). The predicted position and sequence of miR-515-5p binding site on HIF-1α 3`-UTR were displayed in Additional file 1: Figure S2. Consistently, enforced expression of miR-515-5p significantly decreased the luciferase activity of the vector containing wild-type HIF-1α 3`-UTR, and the effect was disappeared after mutation of miR-515-5p binding site (Fig. 4c). Importantly, knockdown of circ-MAT2B dramatically reduced HIF-1α mRNA and protein levels, whereas silencing of miR-515-5p partly rescued HIF-1α expression (Fig. 4d, e). Besides, HIF-1α was substantially elevated in GC tissues as compared to normal tissues (Fig. 4f). Functionally, HIF-1α overexpression significantly rescued the weakened aggressive phenotype of GC cells caused by circ-MAT2B depletion (Fig. 4g, h). These data indicate that HIF-1α is the functional target of the circ-MAT2B/miR-515-5p axis.

### Circ-MAT2B is elevated by HIF-1α

**Fig. 5:**
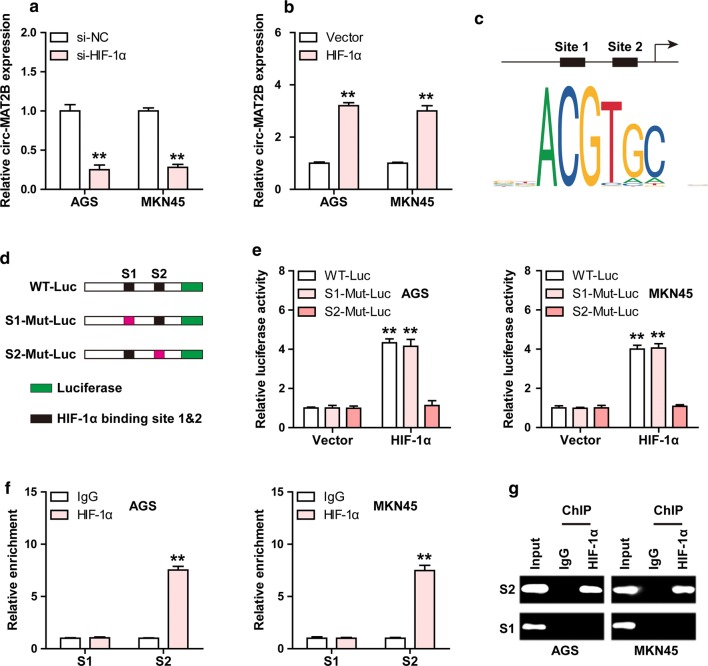
HIF-1α in turn regulates circ-MAT2B. **a**, **b** qRT-PCR analysis of circ-MAT2B expression in GC cells after alternation of HIF-1α level. **c** Two HIF-1α binding motif on circ-MAT2B promoter predicted by the JASPAR database. **d**, **e** The luciferase reporter assay in AGS and MKN45 cells co-transfected with wild-type or mutant circ-MAT2B promoter luciferase vector and control or HIF-1α expression vector. **f, g** The determination of HIF-1α binding on circ-MAT2B promoter using ChIP assay with anti-HIF-1α antibody, followed by PCR analysis. **p < 0.01

Given that HIF-1α is an important transcription factor, we then tested whether circ-MAT2B was regulated by HIF-1α. As shown in Fig. 5a, b, silencing of HIF-1α reduced, while overexpression of HIF-1α increased circ-MAT2B expression in both AGS and MKN45 cells. Through analyzing the JASPAR database, two HIF-1α binding sites (site 1: -1165 ~ -1156, ttacgtgggg; site 2: -324 ~ -315, caacgtgggt) were found on circ-MAT2B promoter (Fig. 5c). We then mutated these two sites and performed luciferase reporter assays, the results showed that HIF-1α overexpression notably increased the activity of circ-MAT2B promoter, whereas mutation of site 2, not site 1, blocked this effect (Fig. 5d, e). To test whether HIF-1α directly bound to circ-MAT2B promoter, ChIP-PCR assay was carried out and the results displayed that HIF-1α was abundantly enriched on site 2, not site 1 (Fig. 5f, g). In addition, hypoxia increased the occupation of HIF-1α on site 2 (Additional file 1: Figure S3).

### Depletion of circ-MAT2B delays tumor growth

**Fig. 6:**
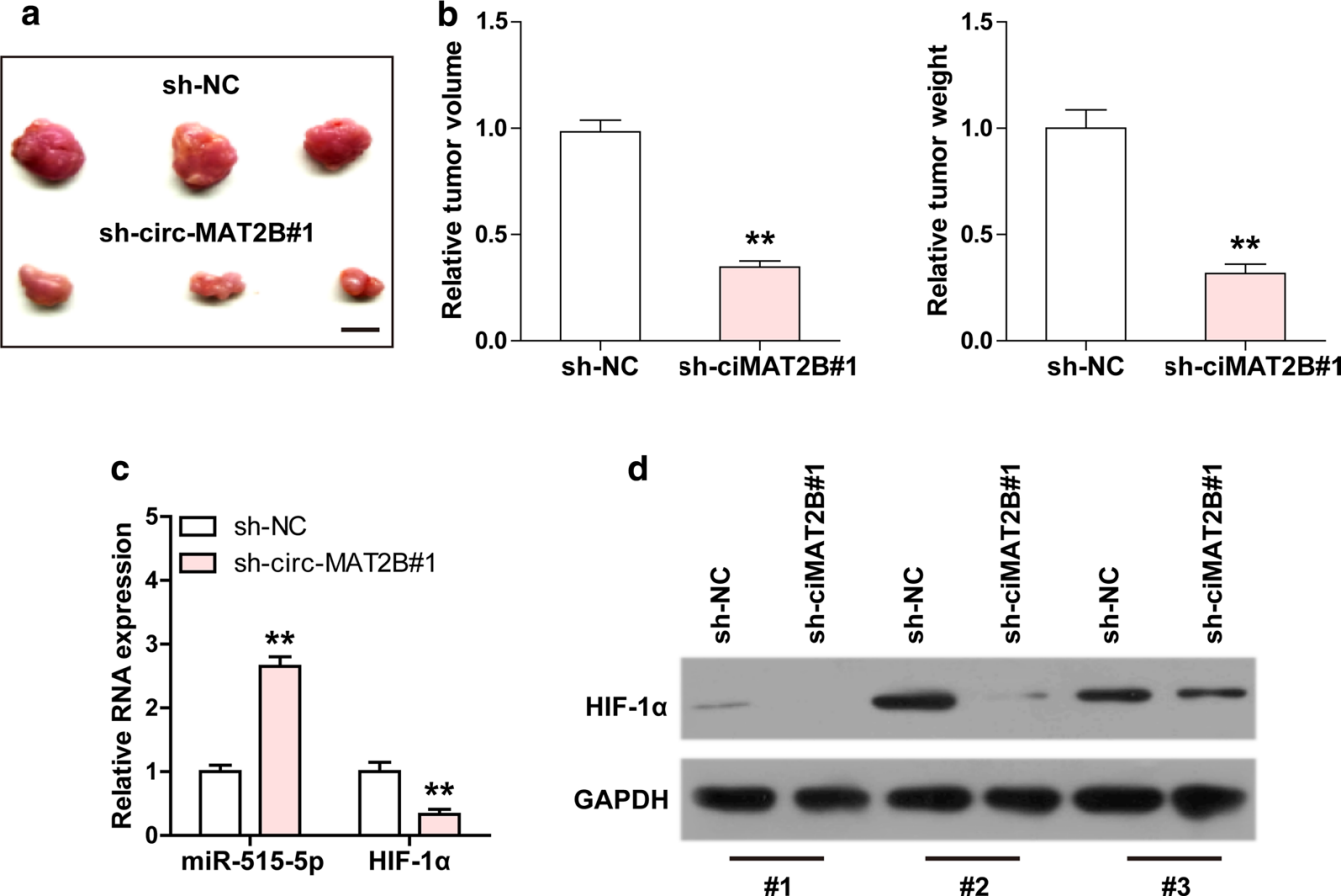
Depletion of circ-MAT2B retards tumor growth in xenograft tumor. **a**, **b** The image and histogram showing the xenograft tumor in control or circ-MAT2B knockdown group. Scale bar = 1 cm, **c** qRT-PCR analysis of miR-515-5p and HIF-1α expression in nude mice bearing xenograft tumor with or without circ-MAT2B knockdown. **d** Western blot analysis of HIF-1α protein expression in nude mice bearing xenograft tumor with or without circ-MAT2B knockdown. GAPDH was used as loading control. **p < 0.01

Lastly, we established xenograft tumor model to test whether circ-MAT2B also functioned in vivo. The results showed that knockdown of circ-MAT2B remarkably reduced tumor volume and weight (Fig. 6a, b). Importantly, miR-515-5p expression was increased, while HIF-1α expression was decreased in circ-MAT2B-depleted group in comparison to control group (Fig. 6c, d). These results suggest that circ-MAT2B knockdown inhibits tumor growth in vivo, which is consistent with the data in vitro.

## Discussion

**Fig. 7:**
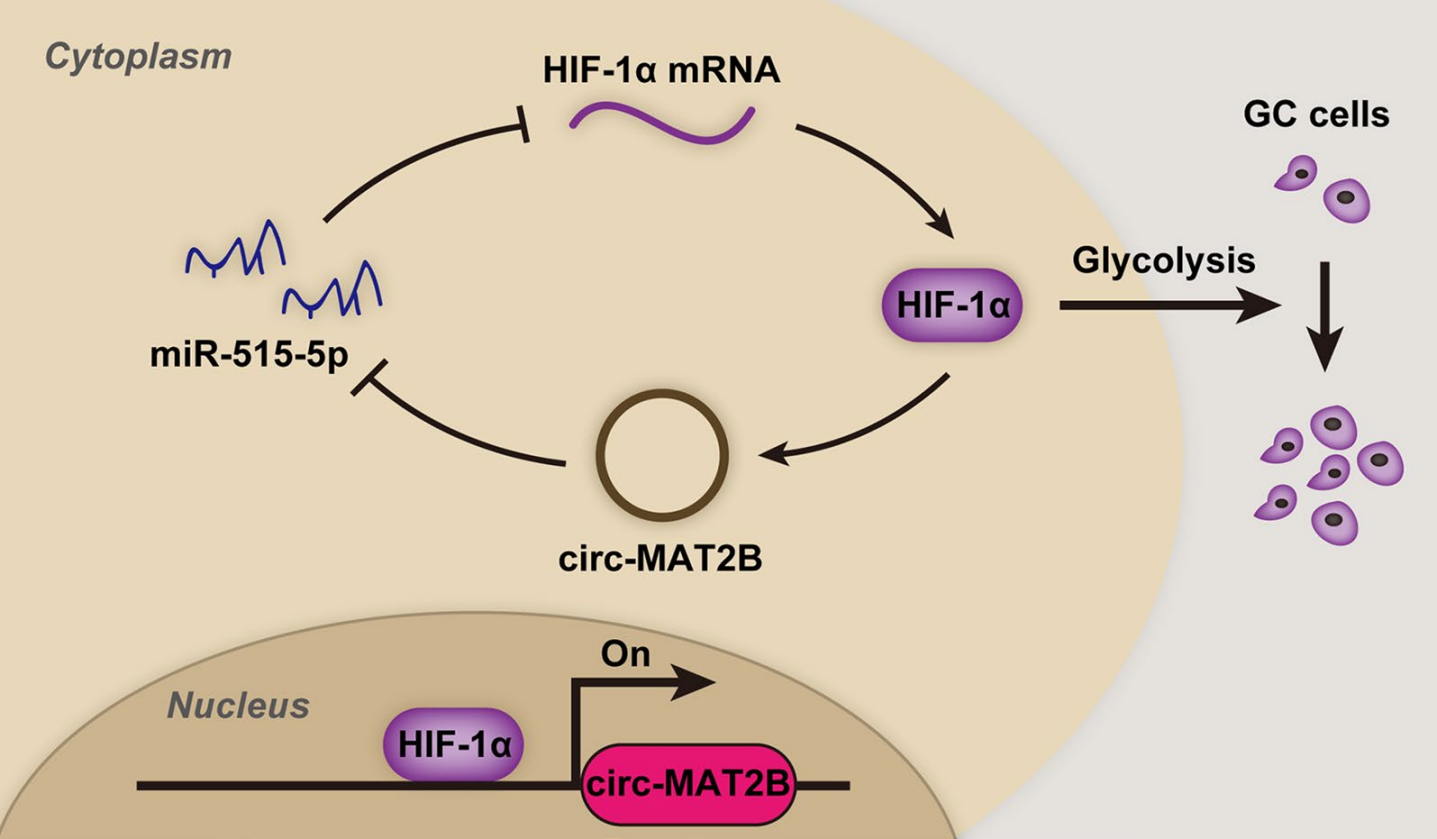
The proposed functional model showing the oncogenic role of circ-MAT2B/miR-515-5p/HIF-1α feedback axis in GC tumorigenesis and glycolysis

In the present study, we identified a glycolysis-associated circRNA in GC. Circ-MAT2B was markedly upregulated in GC plasma and tissue samples, which was closely linked to aggressive clinicopathological features as well as unfavorable prognosis. Functional assays showed that circ-MAT2B was a promoter of GC cell glycolysis and growth both in vitro and in vivo. Regarding the mechanism, circ-MAT2B could elevate HIF-1α expression by abundantly sponging miR-515-5p. In turn, HIF-1α was capable to physically bind to circ-MAT2B promoter and increase circ-MAT2B transcription activity, thus a positive feedback loop was formed between circ-MAT2B and HIF-1α, which amplifies the oncogenic effect of circ-MAT2B in GC (Fig. 7). Our findings provide evidence showing the essential biological relevance of circRNA in cancer, and uncover that deregulation of circ-MAT2B may be responsible for GC malignant progression.

Glucose metabolism reprogramming, a well-established characteristic of multiple cancers, demands a higher rate of glycolysis to meet the increasing demands for cancer cell rapid proliferation [23]. HIF-1α is a well-known driver of cancer cell glycolysis and uncontrolled growth that activates various oncogenic pathways [24]. Studies have shown that HIF-1α is frequently aberrantly expressed in human cancer, and its expression level is tightly regulated by multiple factors at the transcriptional or post-transcriptional level [25]. Here, we verified the upregulation of HIF-1α in GC and further revealed that its upregulation was attributed to circ-MAT2B overexpression. Exogenous expression of HIF-1α could effectively rescued the weakened glycolysis caused by circ-MAT2B knockdown, suggesting that HIF-1α is required for circ-MAT2B function in GC. Further, we found that circ-MAT2B increased HIF-1α expression in an indirect manner, namely the ceRNA mechanism, in which circ-MAT2B was capable to abundantly sponge miR-515-5p and alleviate the inhibitory effect of miR-515-5p on HIF-1α, thereby upregulating HIF-1α. Of note, circ-MAT2B was recently proposed to sponge miR-338-3p in hepatocellular carcinoma [15], however, our preliminary data showed that circ-MAT2B could not absorb miR-338-3p (data not shown), but miR-515-5p in GC, indicating the cell-, tissue- or developmental-stage specific pattern of circRNA function [26]. Further investigation is warranted to clarify the molecular function and biological role of circ-MAT2B in other malignancies.

HIF-1α is a well-documented transcription factor that modulates the expression of a large number of genes via binding to the hypoxia-response element (HRE) located on their promoters [27]. Up to now, many ncRNAs have been reported to be transcriptionally regulated by HIF-1α, such as miR-221 [28], microRNA-23a [29], FOXD2-AS1 [30], LncHITT [31], etc. Nevertheless, there is no evidence showing that HIF-1α can regulate circRNA transcription. In the present study, two HREs were found on circ-MAT2B promoter, and the luciferase report and ChIP-PCR assays revealed that HIF-1α was able to directly bind to the HRE proximal to the transcription start site of circ-MAT2B promoter, resulting in activating circ-MAT2B transcription. Thus, a positive regulatory circuit was formed between HIF-1α and circ-MAT2B, which amplified the oncogenic effect of circ-MAT2B in GC. It will be of great interest to elucidate whether this axis also exists in other malignant tumors.

This work should be considered in light of some limitations. The major disadvantage is that it includes only retrospectively collected samples and information, which may have potential deviation from variable treatments.

## Conclusion

Taken together, our data provide the first evidence that circ-MAT2B functions as an oncogene in GC by facilitating HIF-1α-dependent glycolysis, and also identify circ-MAT2B as a promising diagnostic and prognostic biomarker of GC. Targeting circ-MAT2B and its related regulatory axis implicates the therapeutic possibility for GC patients.

## Supplementary information

**Additional file 1: Figure S1.** The positions and sequences of two miR-515-5p binding sites on circ-MAT2B. **Figure S2.** The position and sequence of miR-515-5p binding site on HIF-1α 3`-UTR. **Figure S3.** The ChIP-qPCR assay using HIF-1α antibody in AGS and MKN45 cells with 1% O_2_. ***p < 0.001. IgG was used as the negative control.

## Data Availability

Please contact authors for data request.

## Abbreviations

CircRNAs: Circular RNAs
GC: Gastric cancer
ceRNA: Competing endogenous RNA
miRNAs: microRNAs
FISH: Fluorescence in situ hybridization
RIP: RNA immunoprecipitation
ChIP: Chromatin immunoprecipitation
ROC: Receiver operating characteristic
