# Supplementary material for: Circular RNA circ-MAT2B facilitates glycolysis and growth of gastric cancer through regulating the miR-515-5p/HIF-1α axis
Source: Cancer Cell Int. 2020 May 16;20:171. doi: 10.1186/s12935-020-01256-1 (PMC7231419; doi:10.1186/s12935-020-01256-1)
Supplement: Supplementary file 1 — Additional file 1: Figure S1. The positions and sequences of two miR-515-5p binding sites on circ-MAT2B. Figure S2. The position and sequence of miR-515-5p binding site on HIF-1α 3`-UTR. Figure S3. The ChIP-qPCR assay using HIF-1α antibody in AGS and MKN45 cells with 1% O2. ***p < 0.001. IgG was used as the negative control. [file 12935_2020_1256_MOESM1_ESM.doc]

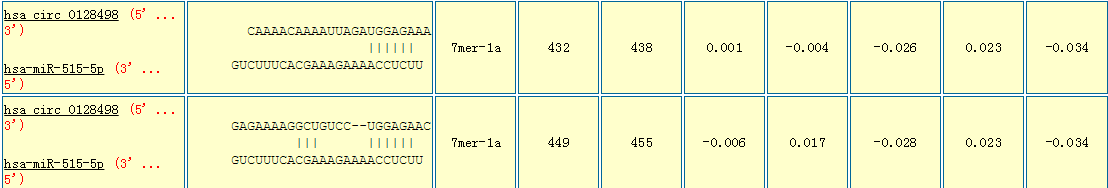

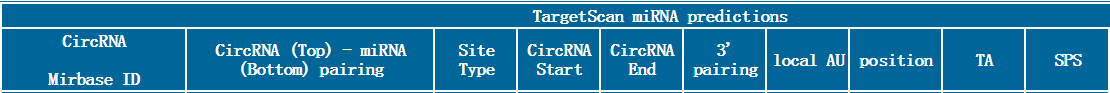


Figure S1. The positions and sequences of two miR-515-5p binding sites on circ-MAT2B.


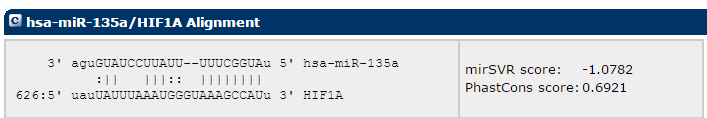


Figure S2. The position and sequence of miR-515-5p binding site on HIF-1α 3`-UTR.


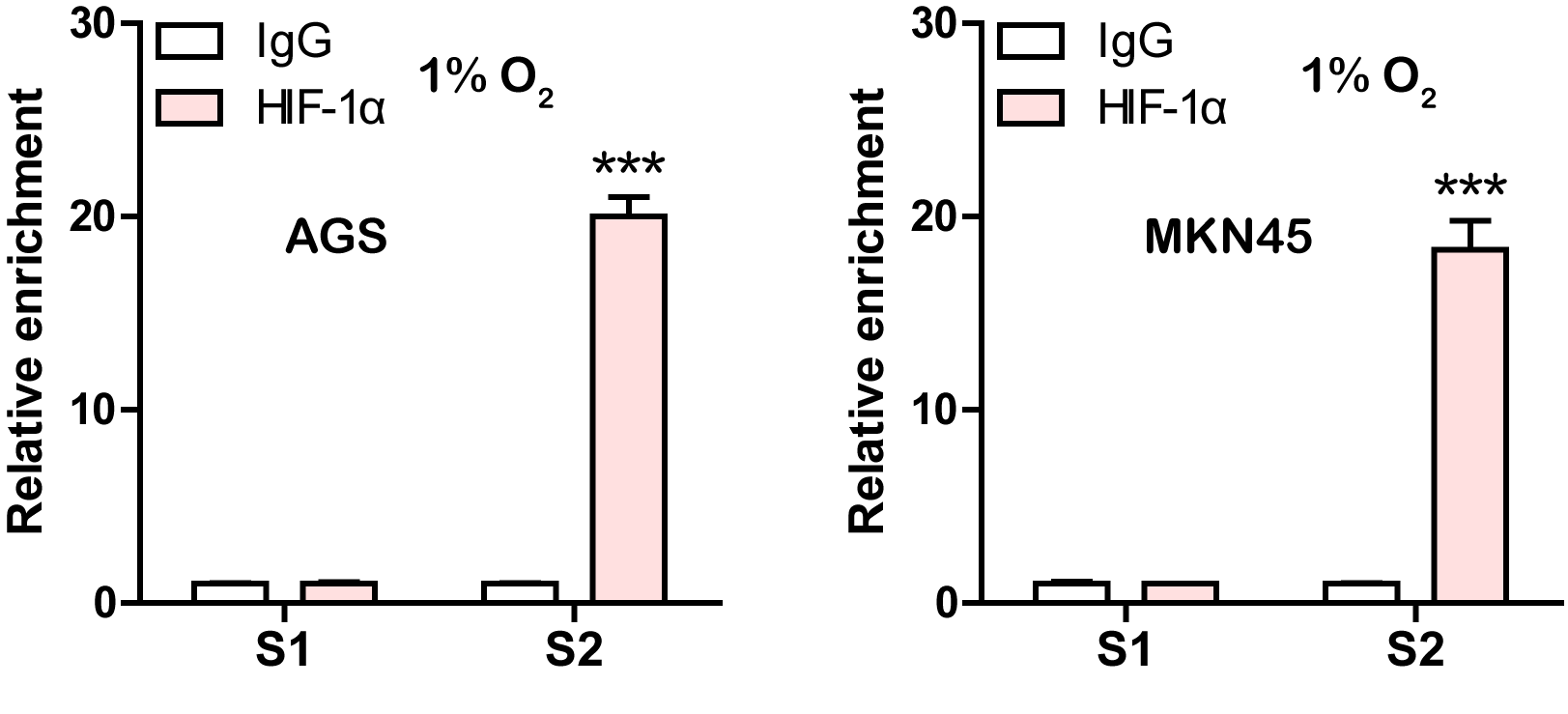


Figure S3. The ChIP-qPCR assay using HIF-1α antibody in AGS and MKN45 cells with 1% O_2_. ************p* < 0.001. IgG was used as the negative control.
